# Prevalence of *Batrachochytrium dendrobatidis* in Amphibians in Northwestern Italy’s Protected Areas

**DOI:** 10.3390/ani15020157

**Published:** 2025-01-09

**Authors:** Arianna Meletiadis, Matteo Riccardo Di Nicola, Stefano Bovero, Marco Favelli, Marzia Pezzolato, Stefania Grella, Giusi Rezza, Pier Luigi Acutis

**Affiliations:** 1 Istituto Zooprofilattico Sperimentale del Piemonte, Liguria e Valle d’Aosta, Via Bologna 148, 10154 Turin, Italy; arianna.meletiadis@izsplv.it (A.M.); marzia.pezzolato@izsplv.it (M.P.); pierluigi.acutis@izsplv.it (P.L.A.); 2 N.G.O. Zirichiltaggi Sardinia Wildlife Conservation, 07100 Sassari, Italy; stefano.bovero@gmx.us (S.B.); yattaran@libero.it (M.F.); 3 Ente di Gestione delle Aree Protette dei Parchi Reali, Via Carlo Emanuele II, 256, 10078 Venaria Reale, Italy; stefania.grella@parchireali.to.it (S.G.); giusi.rezza@parchireali.to.it (G.R.)

**Keywords:** animal diseases, Anurans, chytridiomycosis, emerging infectious diseases, EIDs, La Mandria, *Pelophylax*, Piedmont, Vauda

## Abstract

Chytridiomycosis, caused by the fungus *Batrachochytrium dendrobatidis* (Bd), is a significant threat to global amphibian populations, leading to widespread declines and extinctions. In the spring of 2023, Bd presence was detected in different amphibian species within two protected areas near Turin, Piedmont, Italy, following an unusual mortality event among the common toad (*Bufo bufo*). Histological and molecular analyses confirmed Bd infection in a deceased *Pelophylax* sp. specimen, prompting further investigation. Sampling of 166 individuals across seven amphibian taxa revealed an overall Bd occurrence of 38.6%, with *Pelophylax* sp. showing the highest detection rate (50.5% of 93 individuals). A marked difference in the positivity rate was observed between the two locations, with La Mandria (67.2% of 58) exhibiting significantly higher rates than Vauda (22.9% of 35). While Bd was identified in the sampled amphibians, the exact cause of the observed mortality remains unclear and may involve other pathogens or multifactorial causes, including but not limited to Bd. These findings represent the first documented case of Bd presence in Piedmont after an 18-year gap, highlighting the potential influence of local environmental factors on infection dynamics. The study emphasises the need for expanded, standardised field sampling and further investigation into the various factors affecting amphibian health to guide conservation efforts for vulnerable amphibian species.

## Simple Summary

Amphibians around the world are at risk due to a deadly fungal infection caused by *Batrachochytrium dendrobatidis* (Bd). This study reports a relevant Bd detection in amphibians within two protected areas in northwestern Italy, specifically near Turin. The investigation was prompted by an unusual number of common toad deaths, which led to further analysis of other amphibians sampled in the area. Testing revealed that Bd was present in 38.6% of the sampled amphibians, with higher positivity rates in La Mandria Park compared to the Vauda protected area. However, while Bd was identified, the exact cause of the observed mortality remains uncertain and may involve other pathogens or multifactorial causes. This represents the first reported case of Bd detection in Piedmont in 18 years, underscoring the ongoing threat this pathogen poses to local wildlife. The results highlight the need for comprehensive monitoring and conservation efforts to protect vulnerable amphibian species from Bd and other potential threats.

## 1. Introduction

According to the International Union for Conservation of Nature and Natural Resources [1], amphibians are the most endangered animal class, with 41% of its species currently under threat, indicating the severe risk of biodiversity loss that currently affects the globe [1]. Among the several factors threatening amphibians, those related to human activity are the most serious in terms of the number of affected species [2]. Agriculture, deforestation, and infrastructure construction are the top three activities, impacting between 79% and 43% of species [1,2]. Animal diseases and climate change are the next most significant causes of biodiversity loss, affecting 29% of species [2]. Despite diseases no longer being the primary threat to amphibian species, at the end of the 20th century, a disease caused by the fungus *Batrachochytrium dendrobatidis* (Bd) emerged as a major threat, causing epidemics worldwide, leading to population decline and species extinction [3,4].

This fungus belongs to the genus *Batrachochytrium* (which also includes another species, *B. salamandrivorans*, which is highly pathogenic to Caudata amphibians) and has been observed to infect over 1000 species of amphibians across different continents [5]. Its life cycle consists of two main phases: the motile, waterborne zoospore stage and the stationary zoosporangium stage. Zoospores are uniflagellate, allowing them to move in moist environments, such as amphibian skin or water, to locate a host. Upon contact with the host, the zoospores encyst and penetrate the superficial layers of the skin, where they feed on cellular residues. This leads to the development of the thallic phase, characterised by the formation of zoosporangia within the host’s keratinocytes. Mature zoosporangia release zoospores through discharge tubes projected towards the skin surface. These zoospores can either reinfect the same host, contributing to a local proliferation of the infection or disperse into the environment to infect new hosts [6,7,8]. 

Common clinical signs in amphibians include anorexia, lethargy, abnormal posture, hyperkeratosis, excessive skin shedding, and loss of the righting reflex [7,9,10].

Several authors have observed that the course of the infection varies in different species and populations, depending on environmental and geographical factors that can affect the interaction between the host and the pathogen [5,11,12,13]. Altitude has been identified as one of the main influencing factors [5,12]. Additionally, the optimal environmental temperature range was found to be between 17 °C and 25 °C, and areas with rainfall greater than 150 mm were also identified as contributing factors [5,11,12,13,14,15,16].

It is known that Bd has been present in Europe since at least 1978, following a retrospective study on wild populations of *Bombina variegata pachypus* along the Italian Apennines [14]. In 1999, mass mortalities in the species *Alytes obstetricans*, *Salamandra salamandra*, and *Bufo bufo*, in protected mountain areas of Spain, were reported [15]. Since then, infections have been reported in most European countries (see [11,17,18]). Globally, the IUCN has reported over 50 species as critically endangered or vulnerable due to Bd infection [1].

In Italy, the presence of Bd has been found in multiple areas and in several amphibian species, and it is believed to be the cause of the decline of the endemic species *Euproctus platycephalus* in Sardinia [13,14,16,19,20]. Despite its known presence in the national territory, data concerning its prevalence in various regions are still scarce. In Piedmont, North-western Italy, available data date back more than fifteen years. In 2006, the first documented cases of infection occurred in 1 adult and 36 larvae of the allochthonous *Lithobates catesbeianus* from an area near Turin and Valfenera, Asti [21]. Subsequently, the microorganism was also found in wild populations of *Pelophylax* kl. *esculentus*, from Pianalto (Poirino, TO), from the lakes of Cantarana, Cascina Belvedere, Bric del Papa, and Tetti Brossa, with a prevalence ranging from 10% to 43.7% [22].

This cross-sectional study reports on the presence of Bd in amphibians within two protected areas near the city of Turin, Piedmont, which has a temperate climate featuring relatively evenly distributed rainfall throughout the year, peaking in late spring and autumn. The study was prompted by an event in the spring of 2023, during the breeding season for many local amphibian species, when an unusual mortality event involving common toads (*Bufo bufo*) was reported in a wetland within the Natura 2000 Site of Community Importance (SCI) IT1110079—La Mandria (Venaria Reale, Turin, Piedmont). Following this occurrence, for which no samples were collected, an adult male green frog (*Pelophylax* sp.) found dead in the same site was first sent in an alcohol solution to the laboratories of the Experimental Zooprophylactic Institute of Piedmont, Liguria and Valle d’Aosta in Turin. Subsequently, in light of the results from the green frog sample analysis (see below), the IT1110079—La Mandria and IT1110005—Vauda Natural Park Authorities authorised multiple samplings across the two park wetlands to assess the Bd spread across the territory.

## 2. Materials and Methods

### 2.1. Study Area

**Figure 1:**
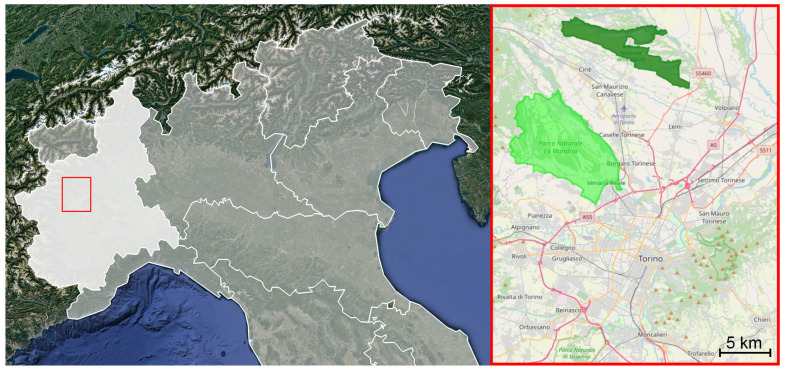
Map of Northern Italy. On the left, the red rectangle highlights the area of Piedmont (region coloured in dense white) where the two Natural Parks involved in the study are located. On the right, in detail, is the location of La Mandria (light green) and Vauda (dark green) to the north of the city of Turin. Map credits: left—Google Earth [Data SIO, NOAA, U.S. Navy, NGA, GEBCO; Image Landsat/Copernicus], modified; right—OpenStreetMap contributors, available under the Open Database License.

IT1110079—La Mandria and IT1110005—Vauda are two protected areas, both located north of Turin (respective coordinates: 45.163, 7.574 and 45.260, 7.674; average altitude: 330 m a.s.l.), at distances of approximately 15 km (La Mandria) and 20 km (Vauda) from the city centre (Figure 1). They are separated by a band approximately 5 to 10 km wide, which consists of flatlands, agricultural fields, small municipalities, and patches of natural vegetation. Additionally, the Stura di Lanzo River runs through the northeastern part of La Mandria, contributing to the natural boundary between the two parks.

Established in 1978, the Parco Naturale La Mandria spans 65.7 km^2^ and is located on the ancient alluvial fan of the Stura di Lanzo River, characterised by Pleistocene fluvio-glacial deposits overlaid by loess from the last glacial period. This geological history has sculpted a terraced landscape, supporting the park’s diverse ecosystems through its unique geomorphology. The terraced plains, predominantly covered with silty loams, showcase the area’s dynamic geological past and ancient erosive processes. Dominated by a climax community of deciduous oak forests, primarily composed of English oak (*Quercus robur*) and hornbeam (*Carpinus betulus*), the park also features interspersed man-made lakes and wetlands.

Established in 1993, the Riserva Naturale della Vauda spans 26.3 km^2^ and is located in the lower Canavese area. Similarly to La Mandria, Vauda is characterised by an ancient landscape of Pleistocene deposits. This historical sedimentation has formed a unique topography of terraces and small valleys, the remains of an ancient alluvial cone from the last ice age, defining the area’s geomorphology. The plains, as in La Mandria, are mainly covered with silty loams and clay, evidencing a dynamic geological past marked by erosive processes. Vauda is dominated by a semi-natural heathland ecosystem, primarily composed of heather (*Calluna vulgaris*) and grasses, interspersed with wetlands that contribute significantly to the area’s biodiversity.

### 2.2. Field Sampling and Molecular Analyses

To screen the highest number of individuals within a limited time frame, an opportunistic, non-standardised sampling was conducted by two of the authors (S.B. and M.F.) from May to July 2023 in both La Mandria and Vauda protected areas. The following amphibian taxa were sampled: *Bufo bufo*, *Hyla intermedia*, *Pelophylax* sp., *Rana* sp., *Rana dalmatina*, *Lissotriton vulgaris*, *Triturus carnifex*, and *Salamandra salamandra*. The screening included larvae, juveniles, and adults of both sexes. The sampling, aimed at the molecular detection of Bd, involved swabbing the entire body surface of the animals, with particular attention to the axillary, inguinal, and ventral regions, using dry swabs that were subsequently stored frozen. For larval stages, swabbing was concentrated on the buccal area as recommended in WOAH Chapter 2.1.1 [23]. In the absence of clinical signs, skin tissue was not collected to minimise the invasiveness of this preliminary screening.

Molecular analyses to detect Bd were first performed on a skin sample from the first green frog received in the laboratory and subsequently on dry swabs from additional samples. All samples were tested using Boyle’s real-time PCR protocol as a qualitative assay [23,24]. DNA was extracted using the Wizard^®^ Genomic DNA Purification Kit (Promega Corp., Madison, WI, USA) following the manufacturers’ protocol. The Real-time TaqMan PCR assay employed the forward primer ITS1-3 Chytr, reverse primer 5.8S Chytr and the Chytr MGB2 probe. The primers are designed to target the conserved regions 5.8S, 18S and 28S rRNA. Reactions were conducted in a total volume of 25 μL, comprising 12.5 μL of 2× TaqMan Master Mix (Applied Biosystems, Foster City, CA, USA), 900 nM of each primer, 250 nM of the MGB2 probe, and 5 μL of template DNA. PCR cycling conditions were as follows: 50 °C for 2 min, 95 °C for 10 min, followed by 50 cycles of 95 °C for 15 s and 60 °C for 1 min. The first sample yielded amplification after approximately 40 cycles without the use of a positive control. This sample was also analysed using an endpoint TaqMan PCR, yielding a positive result. The endpoint PCR protocol used the same primers ITS1-3 Chytr and 5.8S Chytr, amplifying a 160bp portion. The reaction mix comprised 2.5 μL of buffer, 5 μL of CG/Q solution, 1 μL of dNTPs, 1.5 μL of MgCl_2_, 0.625 μL of each primer, 0.25 μL of Taq, 5 μL of DNA template, and 8.5 μL of pure water. The PCR thermal cycling conditions were as follows: 95 °C for 2 min, followed by 50 cycles of 95 °C for 30 s, 55 °C for 30 s, 72 °C for 30 s, and thereafter a final step of 72 °C for 10 min. This product was sequenced to confirm its identity as *B. dendrobatidis* and subsequently used as a positive control for the real-time TaqMan PCR of dry swabs, which amplified after 35–41 cycles. Each subsequent real-time TaqMan PCR reaction also included a no-template control to monitor potential contamination.

The initial endpoint PCR-positive sample underwent PCR product purification using the ExtractME RNA&DNA kit (Blirt, Qiagen, Hilden, Germany), following the manufacturer’s instructions. Cycle sequencing was performed with the BrilliantDye™ Terminator (v1.1) Cycle Sequencing Kit (NimaGen, BV, Nijmegen, Netherlands). Separate reactions were prepared for primers ITS1-3 and 5.8S. Each reaction contained 1 μL of BrilliantDye Terminator, 3.5 μL of 5× sequencing buffer, 1 μL of primer (5 μM), and 1 μL of purified PCR product, in a final volume of 20 μL. The thermal cycling profile consisted of an initial denaturation at 96 °C for 45 s, followed by 28 cycles of 96 °C for 10 s, 50 °C for 5 s, and 60 °C for 2 min, with a final hold 12 °C ∞. The resulting products were purified with the DyeEx 2.0 Spin Kit (Qiagen) according to the manufacturer’s instructions. Purified products were denatured by adding 10 μL of Hi-Di formamide to 5 μL of purified sequencing product in a sequencing plate, following laboratory-best practises. The sample was analysed using the SeqStudio Genetic Analyzer (4-capillary model, Thermo Fisher, Waltham, MA, USA) with the ShortSeq28 sequencing run mode. The resulting sequence (Table S1) was compared against the NCBI BLASTn database (BLASTn: Basic Local Alignment Search Tool. Available online: https://blast.ncbi.nlm.nih.gov/Blast.cgi (accessed on 9 December 2024)), yielding a 100% match with *B. dendrobatidis* (Table S2; Figure S1). Given the high identity, this sequence was employed as a positive control for subsequent real-time TaqMan PCR analyses of swab samples.

Furthermore, to confirm the positivity of samples that amplified beyond 39 cycles, we also performed the previously described endpoint TaqMan PCR on all the positive samples. The results of this analysis confirmed all the detected positives.

For the first green frog, histological analysis was also performed on a skin fragment taken from a depigmented area of the forelimb, using both haematoxylin/eosin (HE) and Periodic Acid-Schiff (PAS) staining.

## 3. Results

The first adult male green frog found dead in La Mandria showed, upon macroscopic examination, slightly depigmented, circular, and non-coalescent punctuate areas along the lateral folds of the dorsal and dorsocaudal brachial area and on the hand (Figure S2). No other macroscopic lesions were found, and skin samples of the depigmented areas were taken to perform histological and molecular analysis.

**Figure 2:**
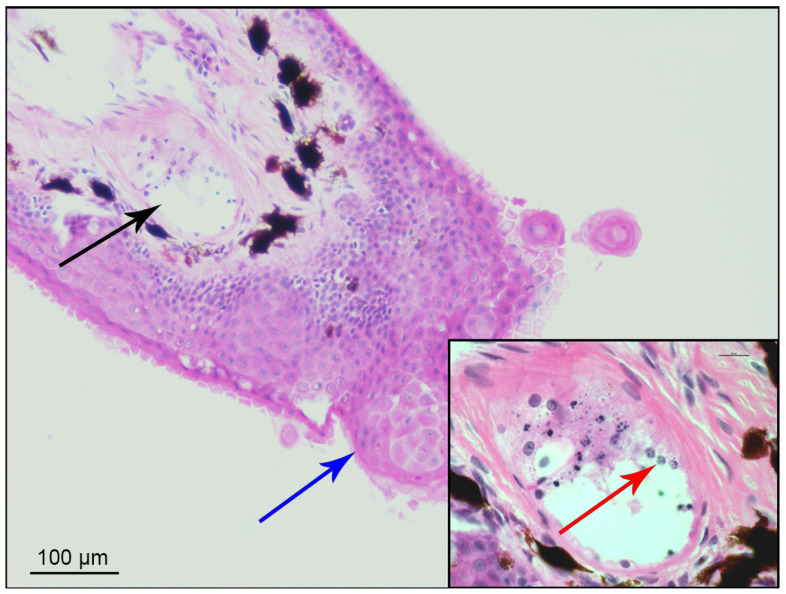
Histological section with PAS staining. Amphibian epidermis affected by *Batrachochytrium dendrobatidis*, exhibiting hyperkeratosis and epidermal hyperplasia with corneal pearl (blue arrow). The black arrow identifies the enlarged area in the insert, where several zoosporangia (red arrow) containing zoospores are visible.

In the histological examination, typical basophilic zoospores with rounded and oval bodies were observed at the epidermal level of a brachial section. Additionally, spherical and oval multifocal formations with papillae protruding to the surface were present in the stratum corneum of the epidermis (Figure 2). Thallic forms of zoosporangia at various stages of development were visible in sections of the skin and subcutis of the lateral folds. The observed pattern was indicative of a fungal infection compatible with Bd.

Skin swabs were collected from a total of 165 animals, all of which showed no gross signs upon macroscopic evaluation, and tested with the above-described PCR protocol, resulting in a total of 166 tested samples. Overall, 64 out of 166 samples (38.6%) were Bd-positive, including 54 out of 125 from La Mandria (43.2%) and 9 out of 40 from Vauda (22.5%). All the collected taxa tested positive except for *Rana* sp. (only three tested animals). Excluding taxa with fewer than 10 individuals sampled, the observed positivity rate per taxon ranged from 15.4% (*Triturus carnifex*) to 50.5% (*Pelophylax* sp., which, with 93 individuals, was also the most sampled taxon) (see Table 1 for the values for each sampled taxon).

**Figure 3:**
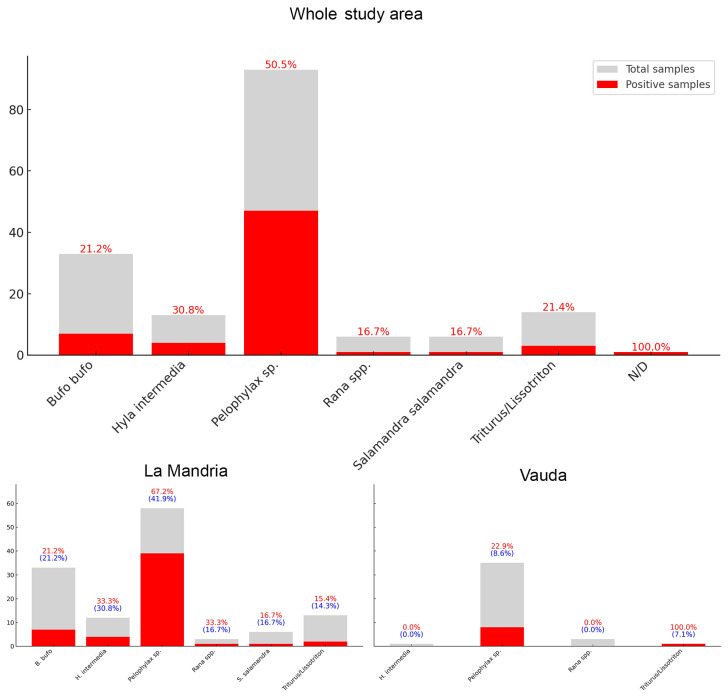
Bd positivity rates after grouping *Rana* spp. and newts to strengthen the analysis (top: overall data, considering the whole study area; bottom left: data related to La Mandria; bottom right: data related to Vauda). Red values: rate of Bd positives relative to the total sample in each specific locality. Blue values in brackets: rate of Bd positives in each locality relative to the total sampled for that taxon across both localities.

For statistical purposes, we considered 165 samples with known taxa. We combined all species of the genus *Rana* and grouped the newts (*L. vulgaris* and *T. carnifex*) to strengthen the analysis due to the limited sample size (see Figure 3). The Bayesian 95% credible intervals (hereafter: CI) were calculated to estimate the overall Bd prevalence and taxon-specific prevalence for the whole study area and location-specific prevalence for *Pelophylax* sp., further stratified by sex and age classes. Statistical significance was inferred based on non-overlapping credible intervals.

The overall Bd prevalence was estimated at 38.3% (CI: 31.2–45.9%). Among the species, *Pelophylax* sp. exhibited the highest prevalence at 50.5% (CI: 40.5–60.4%). Bd estimated prevalence and 95% credible intervals for each taxon are shown in Table 2. Estimated prevalence differed significantly between the two locations for *Pelophylax* sp., with La Mandria showing a value of 66.7% (CI: 54.6–77.8%) compared to 24.3% (CI: 12.2–38.9%) in Vauda.

Regarding sex and age classes, the estimated overall prevalence for *Pelophylax* sp. was 59.2% for females (CI: 40.7–76.6%) and 61.3% for males (CI: 46.6–75.0%), with adults showing a prevalence of 60.9% (CI: 49.4–71.9%), juveniles 50.1% (CI: 2.5–97.6%), and larvae 49.9% (CI: 2.3–97.3%).

In Mandria, the prevalence rates among females and males were 69.2% (CI: 42.9–90.3%) and 65.8% (CI: 50.6–79.5%), respectively. Adults in Mandria showed an estimated prevalence of 67.2% (CI: 54.2–79.4%). The prevalence for juveniles was 49.6% (CI: 2.6–97.7%), and for larvae 50.2% (CI: 2.2–97.6%).

Conversely, in Vauda, females exhibited an estimated prevalence of 50.0% (CI: 26.1–73.6%), while males had a lower prevalence of 20.0% (CI: 0.7–60.0%). Adults had an estimated prevalence of 42.0% (CI: 21.2–64.2%), while the prevalence for juveniles was 50.6% (CI: 2.6–97.5%), and for larvae 49.9% (CI: 2.4–97.6%).

## 4. Discussion

In contrast to the straightforward identification of infections in controlled environments, such as in farmed animals or pets, detecting the mechanisms of infection in natural populations of wild animals is more challenging. In this case, the deaths of several amphibians were observed, and many others tested positive for Bd, suggesting a possible underlying infection.

The Bayesian analysis supports that *Pelophylax* sp. has the highest overall Bd prevalence and reveals significant differences between the surveyed locations, with a higher prevalence in La Mandria compared to Vauda. The consistent Bd prevalence across sexes in Mandria suggests similar exposure or susceptibility levels. In Vauda, the lower prevalence in males compared to females may reflect ecological differences, though non-standardised sampling and small sample size may explain the result. The smaller sample sizes for the other sampled taxa result in wide credible intervals, indicating significant uncertainty in the estimates. These findings highlight the need for increased and balanced sampling, as the limited and uneven sample distribution likely introduces biases affecting the reliability of prevalence estimates. Future research should aim to fill this gap, also extending sampling over a longer period of the year to improve detection rates and provide a more accurate assessment of Bd prevalence in the local amphibian communities.

Costa and colleagues [13] reported an overall Bd prevalence of 6% across Italy, based on a large-scale survey encompassing 1274 individuals from 18 species (13 of which tested positive). This relatively low national prevalence, compared to our findings, especially in La Mandria, suggests that certain local conditions might be facilitating higher prevalence rates. Moreover, Costa et al. emphasised the impact of landscape connectivity and habitat suitability on Bd spread, which was not a direct focus of our study but aligns with the need to understand environmental factors influencing pathogen dynamics. Additionally, it is important to consider the lack of standardisation in sampling efforts across locations, both in terms of total sample size and distribution among species.

As for Piedmont, Federici et al. [22] reported Bd occurrence in *Pelophylax* sp. populations within the Pianalto of Poirino, southeast of Turin, at just 8.5%, with localised peaks of up to 43.7% in some ponds. These values are significantly lower than those observed in La Mandria, suggesting possible differences in local ecological conditions that exacerbate infection rates and/or even a possible intensification of Bd presence over time.

Given the high infectivity of Bd and its ability to infect a wide range of species, it is possible that amphibian populations in nearby areas may also be affected. In particular, chytridiomycosis could threaten populations of rare and protected endemic species listed under Annex II of the European Community’s Habitats Directive. These include *Rana latastei* in the Po River floodplain, *Pelobates fuscus* and *Triturus carnifex* in various lowlands and *Salamandrina perspicillata* in the Piedmont Apennines. Currently, populations of *Salamandra lanzai*, endemic to the Cottian Alps, do not appear to be affected by the pathogen [25].

Potential environmental and climatic factors that might influence Bd prevalence in La Mandria and Vauda include variable seasonal temperatures and precipitation levels, which can create optimal conditions for Bd survival and transmission. Microclimatic conditions in forested or shaded wetlands in these areas may particularly favour Bd, offering cooler and moist habitats. Furthermore, human activities and land use changes around these parks, including agriculture and urban expansion, could be altering local ecosystems in ways that might increase Bd infection risks by affecting water quality and availability or increasing amphibian stress and susceptibility to disease.

The differences between the national, regional, and local findings highlight the complexity of Bd dynamics and suggest that both temporal changes and localised environmental factors may play significant roles in pathogen spread. However, the factors contributing to the differences in Bd prevalence among species and areas at the national level remain hypothetical. Expanding sampling efforts, both in size and temporal coverage, will help in obtaining more reliable and comprehensive data on Bd prevalence and infection, which is essential for the development of effective conservation strategies at both the local and national levels.

## 5. Conclusions

This pilot work was conducted to assess the spread of *Batrachochytrium dendrobatidis* within two protected areas near the city of Turin and allowed us to ascertain a significant occurrence of Bd in populations of various amphibian species. It marks the first documented report from Piedmont, following an 18-year gap in which no infection has been reported in this region.

Notably, the high prevalence of Bd in green frogs from La Mandria Park underscores critical zones for sustained ecological monitoring. Additionally, the possible variation in positivity rates linked to specific environmental conditions calls for a deeper investigation into how these factors influence disease dynamics. Enhancing our understanding of these ecological variables can lead to more precise and effective conservation strategies to protect these vulnerable amphibian communities.

While Bd presence was confirmed, especially in *Pelophylax* sp., we must underscore that the exact cause of the observed mortality in *Bufo bufo* remains inconclusive. Bd presence alone may not fully explain the toad deaths, as other pathogens or multifactorial stressors could have contributed to the mortality event. The interaction between pathogens and other parameters may play a significant role in amphibian health and survival, thus warranting further investigation.

This research, aimed at reporting the spread of Bd in a natural area a few kilometres north of Turin, is of a preliminary nature and presents several limitations. These include the opportunistic nature of the field sampling, resulting in an unbalanced sampling effort and size across species, and the absence of histological verification, which would be useful to determine whether Bd presence is also associated with infection in animals that did not show clinical signs at an initial gross evaluation.

## Figures and Tables

**Table 1:** Sampled amphibian taxa. M = adult male; F = adult female; Ind = adult with undetermined sex; J = newly metamorphosed/juvenile; L = larva; N/A = data not available.

Taxon	Total Bd-Positives (Positivity Rate)	La Mandria Bd-Positives (Positivity Rate)	La Mandria Bd-Positives Age Class/Sex	Vauda Bd-Positives (Positivity Rate)	Vauda Bd-Positives Age Class/Sex
*Rana dalmatina*	1 out of 3 (33.3%)	1 out of 3 (33.3%)	1 J	N/A	N/A
*Pelophylax* sp.	47 out of 93 (50.5%)	39 out of 58 (67.2%)	8 F; 26 M; 1 Ind; 4 J	8 out of 35 (22.9%)	7 F; 1 J
*Hyla intermedia*	4 out of 13 (30.8%)	4 out of 12 (33.3%)	4 M	0 out of 1	N/A
*Bufo bufo*	7 out of 33 (21.2%)	7 out of 33 (21.2%)	5 F; 2 M	N/A	N/A
*Triturus carnifex*	2 out of 13 (15.4%)	2 out of 13 (15.4%)	1 F; 1 M	N/A	N/A
*Rana* sp.	0 out of 3	N/A	N/A	0 out of 3	N/A
*Salamandra salamandra*	1 out of 6 (16.7%)	1 out of 6 (16.7%)	1 L	N/A	N/A
*Lissotriton vulgaris*	1 out of 1 (100%)	N/A	N/A	1 out of 1 (100%)	1 J
N/D	1 out of 1 (100%)	N/A	N/A	N/A	N/A
Total	64 out of 166 (38.6%)	54 out of 125 (43.2%)	14 F; 33 M; 1 Ind; 5 J; 1 L	9 out of 40 (22.5%)	7 F; 2 J

**Table 2:** Bayesian estimated prevalence and 95% credible intervals for Bd presence across grouped taxa. M = adult male; F = adult female; Ind = adult with undetermined sex; J = newly metamorphosed/juvenile; L = larva; N/A = data not available.

Taxon	Total Bd-Positives (Estimated Prevalence and 95% Credible Interval)	La Mandria Bd-Positives (Estimated Prevalence and 95% Credible Interval)	La Mandria Bd-Positives Age Class/Sex	Vauda Bd-Positives (Estimated Prevalence and 95% Credible Interval)	Vauda Bd-Positives Age Class/Sex
*Bufo bufo*	7 out of 33 (22.7%; CI: 10.8–37.7%)	7 out of 33 (22.9%; CI: 10.8–38.0%)	5 F; 2 M	N/A	N/A
*Hyla intermedia*	4 out of 13 (33.4%; CI: 12.5–58.2%)	4 out of 12 (36.0%; CI: 14.2–62.0%)	4 M	0 out of 1 (33.6%; CI: 1.2–84.3%)	N/A
*Pelophylax* sp.	47 out of 93 (50.5%; CI: 40.5–60.4%)	39 out of 58 (66.7%; CI: 54.6–77.8%)	8 F; 26 M; 1 Ind; 4 J	8 out of 35 (24.3%; CI: 12.2–38.9%)	7 F; 1 J
*Rana* spp.	1 out of 6 (25.2%; CI: 3.6–57.9%)	1 out of 3 (40.2%; CI: 6.6–80.4%)	1 J	0 out of 3 (20.0%; CI: 0.7–59.5%)	N/A
*Salamandra salamandra*	1 out of 6 (25.2%; CI: 3.9–58.6%)	1 out of 6 (25.2%; CI: 3.8–59.2%)	1 L	N/A	N/A
*Triturus/Lissotriton*	3 out of 14 (25.1%; CI: 7.9–48.1%)	2 out of 13 (20.0%; CI: 4.7–42.4%)	1 F, 1 M	1 out of 1 (66.4%; CI: 16.0–98.6%)	1 J
Overall	63 out of 165 (38.3%; CI: 31.2–45.9%)	54 out of 125 (43.3%; 35.0–52.0%)	14 F; 33 M; 1 Ind; 5 J; 1 L	9 out of 40 (23.8%; CI: 12.3–37.6%)	7 F; 2 J

## Data Availability

All data generated or analysed during this study are included in this published article.

## Supplementary Materials

The following supporting information can be downloaded at https://www.mdpi.com/article/10.3390/ani15020157/s1. Table S1. Sequence from the first sample; Table S2. BLASTn align results: sequence from the first individual. Query: 602831. Database: core_nt; Figure S1. Distance tree of pairwise results, in yellow query 602831; Figure S2. Adult male *Pelophylax* sp. found dead, shown during macroscopic examination.
