# Supplementary material for: Prevalence of Batrachochytrium dendrobatidis in Amphibians in Northwestern Italy’s Protected Areas
Source: Animals (Basel). 2025 Jan 9;15(2):157. doi: 10.3390/ani15020157 (PMC11758637; doi:10.3390/ani15020157)
Supplement: Supplementary file 1 [file animals-15-00157-s001.zip › animals-3389909-supplementary.pdf]

Prevalence of *Batrachochytrium dendrobatidis* in Amphibians in Northwestern Italy's Protected Areas

Arianna Meletiadis, Matteo Riccardo Di Nicola, Stefano Bovero, Marco Favelli, Marzia Pezzolato, Stefania Grella, Giusi Rezza and Pier Luigi Acutis

Supplementary File

Table S1. Sequence from the first sample.

AGTTGTTTTTAATATTTATTTTCAATTTTTTTCAATTAAATCATTCAATTTCCAATTAATTTGTCGAAAA AATAAATAAATTTTGTTCGACTCGTGACATATGGCACACTGTTTATATGAAGG

Table S2. BLASTn align results: sequence from the first individual. Query: 602831. Database: core_nt.

query	subject acc.ver	% identity	alignment length	mismatches	gap opens	q. start	q. end	s. start	s. end	evalue	bit score
Query_602831	JX993750.1	100.000	111	0	0	1	111	112	2	6.63e-49	206
Query_602831	MG252127.1	100.000	92	0	0	1	92	92	1	2.42e-38	171
Query_602831	MG252076.1	100.000	92	0	0	1	92	92	1	2.42e-38	171
Query_602831	MG252105.1	100.000	92	0	0	1	92	92	1	2.42e-38	171
Query_602831	AB469199.1	100.000	92	0	0	1	92	92	1	2.42e-38	171
Query_602831	MG601126.1	99.099	111	1	0	1	111	112	2	3.09e-47	200
Query_602831	FJ010547.1	99.074	108	1	0	1	108	108	1	1.44e-45	195
Query_602831	HQ176489.1	99.048	105	1	0	1	105	105	1	6.68e-44	189
Query_602831	MH745069.1	99.000	100	1	0	1	100	100	1	4.02e-41	180
Query_602831	MZ520158.1	98.980	98	1	0	1	98	100	3	5.20e-40	176
Query_602831	MG601119.1	98.980	98	0	1	15	111	98	1	1.87e-39	174
Query_602831	KF702314.1	98.936	94	0	1	15	107	94	1	3.13e-37	167
Query_602831	MG252112.1	98.925	93	0	1	1	92	93	1	1.13e-36	165
Query_602831	MK573616.1	98.913	92	1	0	1	92	92	1	1.13e-36	165
Query_602831	MK573579.1	98.913	92	1	0	1	92	92	1	1.13e-36	165
Query_602831	MK573588.1	98.913	92	1	0	1	92	92	1	1.13e-36	165
Query_602831	MK573587.1	98.913	92	1	0	1	92	92	1	1.13e-36	165
Query_602831	MG252120.1	98.913	92	1	0	1	92	92	1	1.13e-36	165
Query_602831	MK573569.1	98.913	92	1	0	1	92	92	1	1.13e-36	165
Query_602831	MK573624.1	98.913	92	1	0	1	92	92	1	1.13e-36	165
Query_602831	JN870758.1	98.913	92	1	0	1	92	92	1	1.13e-36	165
Query_602831	MK573623.1	98.913	92	1	0	1	92	92	1	1.13e-36	165
Query_602831	MK573618.1	98.913	92	1	0	1	92	92	1	1.13e-36	165
Query_602831	MG252123.1	98.913	92	1	0	1	92	92	1	1.13e-36	165
Query_602831	MK573608.1	98.913	92	1	0	1	92	92	1	1.13e-36	165
Query_602831	MK573602.1	98.913	92	1	0	1	92	92	1	1.13e-36	165
Query_602831	MK573620.1	98.913	92	1	0	1	92	92	1	1.13e-36	165
Query_602831	MK573621.1	98.913	92	1	0	1	92	92	1	1.13e-36	165
Query_602831	MK573607.1	98.913	92	1	0	1	92	92	1	1.13e-36	165
Query_602831	MK573594.1	98.913	92	1	0	1	92	92	1	1.13e-36	165
Query_602831	MK573606.1	98.913	92	1	0	1	92	92	1	1.13e-36	165
Query_602831	MG252122.1	98.913	92	1	0	1	92	92	1	1.13e-36	165
Query_602831	MK573597.1	98.913	92	1	0	1	92	92	1	1.13e-36	165
Query_602831	MK573589.1	98.913	92	1	0	1	92	92	1	1.13e-36	165
Query_602831	MK573567.1	98.913	92	1	0	1	92	92	1	1.13e-36	165
Query_602831	JN870763.1	98.913	92	1	0	1	92	92	1	1.13e-36	165
Query_602831	MG252093.1	98.913	92	1	0	1	92	92	1	1.13e-36	165

Query_602831	MK573581.1	98.913	92	1	0	1	92	92	1	1.13e-36	165
Query_602831	MG252121.1	98.913	92	1	0	1	92	92	1	1.13e-36	165
Query_602831	MK573596.1	98.913	92	1	0	1	92	92	1	1.13e-36	165
Query_602831	MG252098.1	98.913	92	1	0	1	92	92	1	1.13e-36	165
Query_602831	OQ275246.1	98.913	92	1	0	1	92	92	1	1.13e-36	165
Query_602831	JN870762.1	98.913	92	1	0	1	92	92	1	1.13e-36	165
Query_602831	MK573622.1	98.913	92	1	0	1	92	92	1	1.13e-36	165
Query_602831	MG252108.1	98.913	92	1	0	1	92	92	1	1.13e-36	165
Query_602831	MK573592.1	98.913	92	1	0	1	92	92	1	1.13e-36	165
Query_602831	MG252099.1	98.913	92	1	0	1	92	92	1	1.13e-36	165
Query_602831	MK573619.1	98.913	92	1	0	1	92	92	1	1.13e-36	165
Query_602831	MK573565.1	98.913	92	1	0	1	92	92	1	1.13e-36	165
Query_602831	MK573610.1	98.913	92	1	0	1	92	92	1	1.13e-36	165
Query_602831	JQ582927.1	98.387	124	2	0	1	124	201	78	8.52e-53	219
Query_602831	MG601127.1	98.198	111	2	0	1	111	112	2	1.44e-45	195
Query_602831	HQ176488.1	98.113	106	1	1	1	105	106	1	3.11e-42	183
Query_602831	HQ176492.1	98.095	105	2	0	1	105	105	1	3.11e-42	183
Query_602831	OR717493.1	97.959	98	1	1	15	111	98	1	8.70e-38	169
Query_602831	MG601118.1	97.938	97	1	1	15	110	97	1	3.13e-37	167
Query_602831	JQ582904.1	97.581	124	3	0	1	124	195	72	3.96e-51	213
Query_602831	JQ582915.1	97.581	124	3	0	1	124	201	78	3.96e-51	213
Query_602831	KX115399.1	97.581	124	3	0	1	124	124	1	3.96e-51	213
Query_602831	KX115404.1	97.581	124	3	0	1	124	124	1	3.96e-51	213
Query_602831	JQ582938.1	97.581	124	3	0	1	124	199	76	3.96e-51	213
Query_602831	NR_119535.1	97.581	124	3	0	1	124	199	76	3.96e-51	213
Query_602831	JQ582937.1	97.581	124	3	0	1	124	200	77	3.96e-51	213
Query_602831	JQ582903.1	97.581	124	3	0	1	124	196	73	3.96e-51	213
Query_602831	AY598034.1	97.581	124	3	0	1	124	124	1	3.96e-51	213
Query_602831	KX115405.1	97.581	124	3	0	1	124	124	1	3.96e-51	213
Query_602831	MT730780.1	97.581	124	3	0	1	124	1976	1853	3.96e-51	213
Query_602831	JQ582921.1	97.581	124	2	1	1	124	200	78	1.43e-50	211
Query_602831	KX115392.1	96.800	125	3	1	1	124	125	1	1.84e-49	207
Query_602831	KX115393.1	96.800	125	2	2	1	124	124	1	1.84e-49	207
Query_602831	JQ582916.1	96.800	125	3	1	1	124	196	72	1.84e-49	207
Query_602831	JQ582905.1	96.774	124	4	0	1	124	201	78	1.84e-49	207
Query_602831	JQ582898.1	96.774	124	4	0	1	124	198	75	1.84e-49	207
Query_602831	KX115397.1	96.774	124	4	0	1	124	124	1	1.84e-49	207
Query_602831	KX115394.1	96.639	119	4	0	4	122	121	3	1.11e-46	198
Query_602831	KU669287.1	94.595	111	6	0	1	111	112	2	1.87e-39	174
Query_602831	JQ582906.1	94.444	108	4	2	19	124	186	79	1.13e-36	165
Query_602831	JQ582923.1	93.846	130	2	1	1	124	208	79	1.86e-44	191
Query_602831	JQ582940.1	93.846	130	2	1	1	124	208	79	1.86e-44	191
Query_602831	JQ582925.1	93.798	129	3	1	1	124	207	79	6.68e-44	189
Query_602831	JQ582935.1	93.798	129	3	1	1	124	207	79	6.68e-44	189
Query_602831	JQ582922.1	93.798	129	3	1	1	124	207	79	6.68e-44	189

Query_602831	MW147112.1	93.701	127	5	2	1	124	127	1	2.40e-43	187
Query_602831	MH745071.1	93.694	111	7	0	1	111	112	2	3.13e-37	167
Query_602831	JQ582941.1	93.651	126	4	3	1	124	198	75	8.64e-43	185
Query_602831	MW147111.1	92.857	126	6	3	1	124	125	1	4.02e-41	180
Query_602831	JQ582902.1	92.063	126	6	4	1	124	198	75	1.87e-39	174
Query_602831	MW147110.1	92.063	126	5	5	1	124	123	1	6.73e-39	172
Query_602831	JQ582926.1	92.000	125	4	2	1	124	198	79	2.42e-38	171
Query_602831	KX115396.1	92.000	125	4	2	1	124	120	1	2.42e-38	171
Query_602831	KX115398.1	92.000	125	4	2	1	124	120	1	2.42e-38	171
Query_602831	KX115402.1	92.000	125	4	2	1	124	120	1	2.42e-38	171
Query_602831	JQ582924.1	92.000	125	4	2	1	124	198	79	2.42e-38	171
Query_602831	JQ582928.1	92.000	125	4	2	1	124	198	79	2.42e-38	171
Query_602831	JQ582920.1	92.000	125	4	2	1	124	198	79	2.42e-38	171
Query_602831	JQ582929.1	92.000	125	4	2	1	124	198	79	2.42e-38	171
Query_602831	JQ582931.1	91.339	127	5	6	1	124	199	76	8.70e-38	169
Query_602831	JQ582918.1	91.339	127	5	6	1	124	199	76	8.70e-38	169
Query_602831	JQ582895.1	91.339	127	5	6	1	124	197	74	8.70e-38	169
Query_602831	JQ582919.1	91.339	127	5	6	1	124	199	76	8.70e-38	169

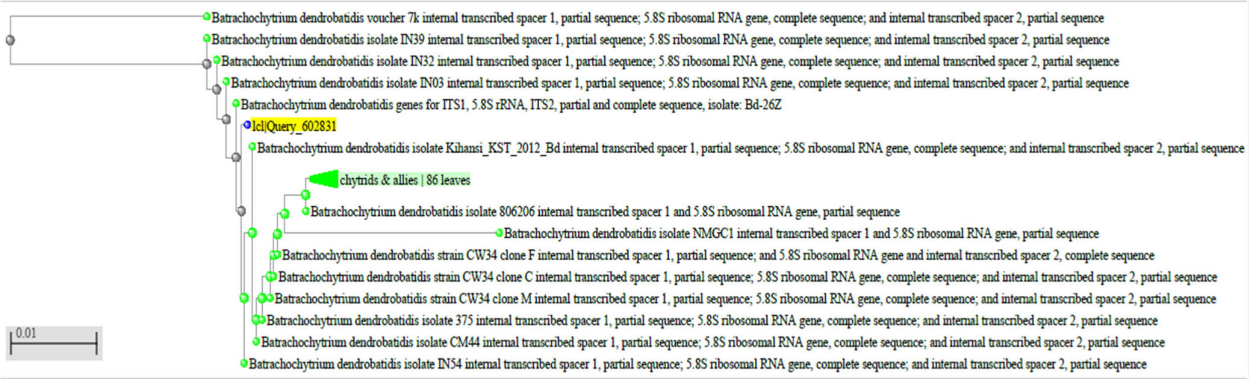

Figure S1. Distance tree of pairwise results, in yellow query 602831.

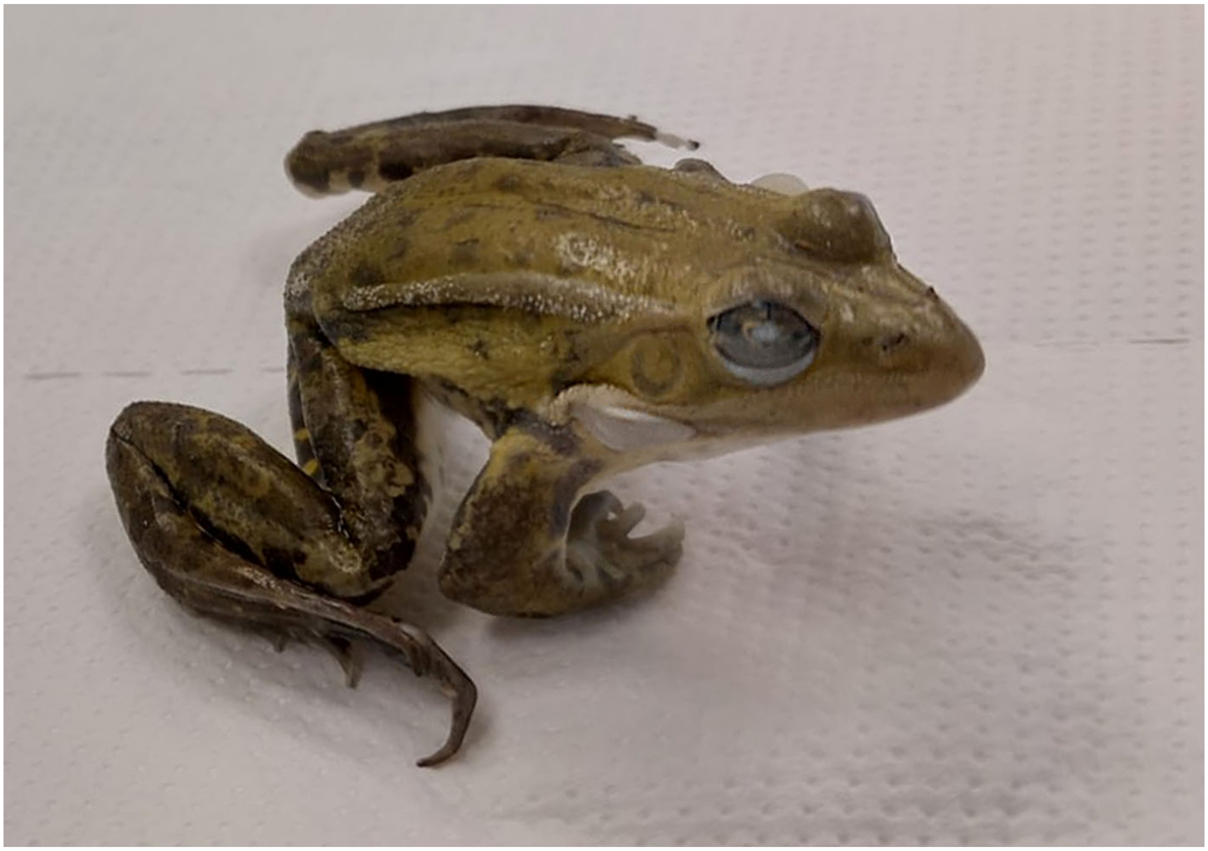

Figure S2. Adult male *Pelophylax* sp. found dead, shown during macroscopic examination.
